# Metastatic Renal Cell Carcinoma to the Cerebrum in a Patient With Lynch Syndrome: A Case Report

**DOI:** 10.7759/cureus.14804

**Published:** 2021-05-02

**Authors:** Jaron M Hrushka, Randall Z Allison

**Affiliations:** 1 Department of Neurosurgery, University of Texas Medical Branch, Galveston, USA

**Keywords:** hereditary nonpolyposis colorectal cancer syndrome, lynch syndrome, metastatic renal cell carcinoma, histopathological diagnosis, intracranial lesion

## Abstract

Hereditary nonpolyposis colorectal cancer (HNPCC) or Lynch syndrome is an autosomal-dominant genetic disorder of DNA mismatch repair associated with many forms of cancer, especially colorectal and including renal cell. In this report, we present a case of a patient with a known history of HNPCC whose first presentation of renal cell carcinoma (RCC) was associated with a symptomatic intracranial lesion. After intracranial imaging, resection, and pathologic examination, the lesion was revealed to be of RCC origin. Further imaging revealed primary RCC. HNPCC may present with neurologic symptoms prior to the diagnosis of primary cancer, and lower levels of suspicion for intracranial lesions may be required to properly treat this patient population.

## Introduction

Brain metastasis in hereditary nonpolyposis colorectal cancer (HNPCC) is a rare occurrence. A literature review has identified only one case of brain metastasis in a patient with HNPCC from primary pancreatic cancer. There are no published reports of HNPCC presenting with brain metastasis from a primary renal cell carcinoma (RCC). In this report, we present a male patient with HNPCC presenting with right upper extremity dyspraxia associated with headache and dizziness and was found to have a left parietal lesion. He subsequently underwent left parietal craniotomy for tumor resection. Histopathologic reports confirmed RCC with paired-box gene 8 (PAX8) and cytokeratin 7 (CK7) staining. Although such a presentation is exceedingly rare, focal neurological deficits manifesting in a patient with HNPCC should raise suspicion for metastasis to the brain.

## Case presentation

History and presentation

**Figure 1:**
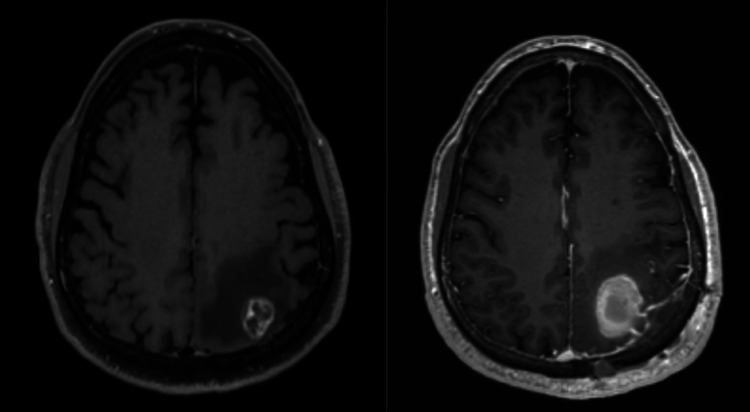
T1 MRI of the patient Preoperative T1 + gad revealing a left parietal 2-cm peripherally enhancing mass (left), and postoperative T1 + gad revealing hemorrhage into the tumor bed with no residual tumor (right) MRI: magnetic resonance imaging; gad: gadolinium

A 64-year-old man presented with right-hand dyspraxia for approximately two days; it was associated with a bitemporal headache and dizziness. He endorsed a 50-lb weight loss over the past year. He had a history of HNPCC with an MLH1 mutation. He had undergone a right hemicolectomy, appendectomy, and cholecystectomy at 47 years of age after a colonoscopy had identified stage I infiltrating moderately differentiated adenocarcinoma in the hepatic flexure of the colon. He had subsequently completed 5‐fluorouracil (5‐FU) and leucovorin therapy. Follow-up colonoscopies had revealed two other polyps, which had been biopsy-proven tubular adenomas. At 49 years of age, he had undergone a partial small bowel resection due to small bowel obstruction and had subsequently developed a moderate ventral hernia. He had an extensive family history of colon and endometrial cancers. He had never smoked cigarettes. A neurological examination revealed a right pronator drift with no associated weakness or cranial nerve abnormalities. An MRI with intravenous gadolinium (gad) demonstrated a peripherally enhancing 2 x 2-cm mass in the left superior parietal lobe with gradient blooming signal causing mass effect on the occipital horn, associated with vasogenic edema (Figure 1).

**Figure 2:**
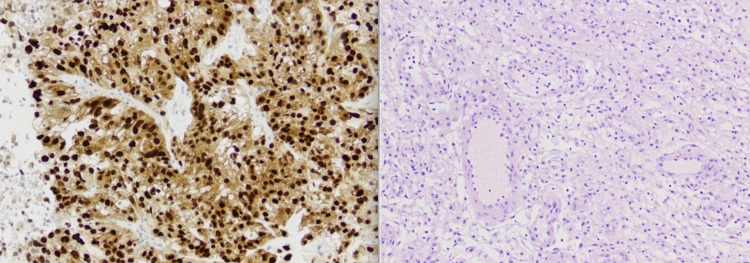
Pathological findings The neoplasm has strong diffuse immunoreactivity to PAX-8, supporting renal cell carcinoma as primary (left), and epithelioid morphology with a moderate degree of pleomorphism and focal areas of clear cell morphology (right) PAX-8: paired-box gene 8

Histopathologic reports confirmed RCC with PAX-8 and CK7 staining (Figure 2).

A CT of the chest, abdomen, and pelvis with Omnipaque revealed a 5.1 x 5.0 x 4.9-cm heterogeneously enhancing mass arising from the lower pole of the right kidney, concerning for RCC, as well as a 3-mm right lower lung lesion.

Operation and postoperative course

A left parietal craniotomy was undertaken with MAYFIELD® Pins (Integra LifeSciences, Plainsboro Township, NJ). A yellow gyrus was easily identifiable adjacent to the vein of Trolard. The vein was mobilized away from the affected gyrus. A cortisectomy was performed and a beefy red tumor was identified immediately deep into the gray matter. A 2 x 2-cm tumor was removed en bloc and sent for both frozen and permanent pathological examination. Postoperatively, the patient was neurologically intact, and a postoperative MRI revealed no evidence of residual tumor, but moderate hemorrhage into the tumor bed was seen (Figure 2). The patient convalesced well in the hospital and was discharged home on postoperative day four. He subsequently underwent single-fraction radiosurgery (SRS) to the tumor cavity with 27 Gy over three fractions. He was staged with T3N0M1 metastatic RCC, with no subtype specified.

## Discussion

HNPCC is an autosomal-dominant inherited cancer syndrome caused by mutations in mismatch repair genes resulting in microsatellite instability. It accounts for between 1.8-2.1% of all colon and endometrial cancers [1]. There is evidence for associations between HNPCC and several other cancers, notably glioblastoma, urothelial cancer, ovarian cancer, sebaceous gland adenomas, keratoacanthomas, and carcinomas of the small bowel [2,3]. One study discovered a statistically significant linkage between HNPCC and RCC in patients aged 50-69 years (IRR=4.99, p<0.05) [4]. Many HNPCC-associated cancers have been heavily linked to specific mutations within the MMR gene. Of the HNPCC patients with RCC, six had an MSH2 mutation, six had MLH1, and one patient had MSH6.

Although RCC found in patients with HNPCC is rare, and metastasis to the brain has not been reported previously, RCC is commonly associated with brain metastasis. A literature review has identified only one case of brain metastasis in a patient with HNPCC from primary pancreatic cancer [5]. In a Surveillance, Epidemiology, and End Results (SEER) analysis published in 2018 identifying 6,328 patients with metastatic RCC, 749 (12.3%) patients had brain metastasis at the time of diagnosis. Of these patients, 147 (19.9%) had isolated brain metastasis as seen in our case [6].

The mainstay of treatment for brain metastasis from RCC is stereotaxic radiosurgery with or without tumor resection, depending on the size and location of the lesion. There is a lack of evidence with regard to which systemic therapies are best suited for this patient population, but anti-vascular endothelial growth factor tyrosine kinase inhibitors sorafenib and sunitinib have shown promise in several clinical trials [7]. Because RCC is not uncommon in HNPCC, it would be reasonable to consider a preoperative angiogram to assess whether preoperative embolization of tumor feeders may decrease the risk of tumor cavity bleeding post tumor resection. 

According to a study analyzing 413 cases of RCC with metastasis to the brain on initial presentation, the mean survival was between 8.6 and 17.7 months depending on the treatment modality. There was an increase in survival in patients who received cytoreductive nephrectomy, a generally accepted relative contraindication for RCC with brain metastasis despite a paucity of data examining its impact [8]. Of note, patients presenting with central nervous system (CNS) symptoms at the time of diagnosis tend to have increased mean survival, likely due to earlier recognition of the condition [9]. Although treatment methods and prognostic indicators have been well described in the general population with RCC metastasis to the brain, its relevance to our patient is difficult to ascertain since studies and clinical trials present in the literature do not include patients with comorbid malignancies.

## Conclusions

HNPCC, a cancer syndrome with a broad tumor spectrum, may present with RCC in rare cases. We described the case of a patient with HNPCC with metastatic RCC to the left parietal lobe, which was treated with surgical resection and subsequent radiotherapy. Although exceedingly uncommon, metastatic RCC to the brain can indeed occur in patients with HNPCC.
